# Structural and Functional Alterations in Hemodialysis Patients: A Voxel-Based Morphometry and Functional Connectivity Study

**DOI:** 10.3389/fnhum.2020.00080

**Published:** 2020-03-11

**Authors:** Mei Jin, Liyan Wang, Hao Wang, Xue Han, Zongli Diao, Wang Guo, Zhenghan Yang, Heyu Ding, Zheng Wang, Peng Zhang, Pengfei Zhao, Han Lv, Wenhu Liu, Zhenchang Wang

**Affiliations:** ^1^Department of Radiology, Beijing Friendship Hospital, Capital Medical University, Beijing, China; ^2^Department of Nephrology, Faculty of Kidney Diseases, Beijing Friendship Hospital, Capital Medical University, Beijing, China

**Keywords:** end-stage renal disease, hemodialysis, voxel-based morphometry, functional connectivity, thalamus, caudate

## Abstract

Structural and functional brain alterations have been always observed in end-stage renal disease (ESRD) patients undergoing hemodialysis. The present study aimed to investigate the gray matter volume (GMV) changes in hemodialysis patients compared with those noted in healthy subjects, as well as explore the associated functional connectivity alterations based on the abnormal GMV regions. The experiments revealed the effects of regional morphometry aberrance on the brain functional integrity. A total of 46 hemodialysis patients (53.11 ± 1.58 years, 28 males) and 47 healthy subjects (55.57 ± 0.86 years, 22 males) were enrolled in the present study. All subjects underwent high-resolution T1-weighted imaging, resting-state functional MR imaging, and laboratory examinations were performed in hemodialysis patients. The GMV deficits were analyzed using voxel-based morphometry (VBM) and regions with GMV alteration were defined as seeds for functional connectivity analysis. Correlation analyses between significantly different regions and the results of the blood examination were further performed. We found that bilateral thalamus exhibited significantly increased volumes in the hemodialysis patients compared with those of the healthy subjects. However, the bilateral rectus, bilateral caudate, and bilateral temporal gyrus demonstrated significantly decreased volumes. When the regions with GMV alterations were defined as seeds, the hemodialysis patients exhibited decreased integrations in the thalamo-cortical network and within the basal-ganglia connection. The present study revealed the presence of different types of structural and functional brain impairments in hemodialysis patients.

## Introduction

End-stage renal disease (ESRD) is known as stage 5 of chronic kidney disease with almost complete reduction of a glomerular filtration rate (<15 ml/min/1.73 m^2^) (Brouns and De Deyn, 2004; Foley and Collins, 2007). ESRD patients have to undergo alternative treatment, such as hemodialysis, peritoneal dialysis, and renal transplantation (Foley and Collins, 2007). ESRD is a chronic renal disease with cerebral structural and functional alterations due to the “kidney-brain axis” (Masaki and Masatsugu, 2010; Bugnicourt et al., 2013). For example, cerebrorenal interaction between poor renal function and brain impairment is based on the anatomy or connection between the cerebrum and the glomerulus. Vasoregulatory similarity has been performed in terms of the common pathogenetic basis of ESRD. These may be involved in the “kidney–brain axis,” resulting in multiple clinical manifestations. Cognitive dysfunction involving a range of cognitive regions is highly prevalent in hemodialysis patients and may be associated with attention, memory, and planning. The early detection of the cognitive deficits of hemodialysis patients can aid the understanding of the potential neuronal mechanisms and lead to timely interventions that may reduce frequent hospitalizations, the time duration spent in hospital, and even morbidity incidence (Qiu et al., 2014).

Recently, structural and functional neuroimaging techniques are considered as major and promising tools playing an important role in the identification of cerebral abnormalities in patients with brain disease (Guo et al., 2012; Liu et al., 2015). With regard to hemodialysis patients, structural imaging techniques, such as diffusion-tensor imaging (Chou et al., 2019), have been used to investigate white matter integrity. Functional imaging techniques, such as positron emission tomography (Bural et al., 2018), single-photon-emission computed tomography (Polinder-Bos et al., 2018), magnetic resonance spectroscopy (Zhang et al., 2017), and arterial spin labeling (Zheng et al., 2016) have been used to measure cerebral functional abnormalities and metabolic impairments in ESRD patients. However, these results have been inconsistent due to the diverse population cohorts and different methodologies used. Moreover, resting-state functional MRI has the ability to detect spontaneous fluctuations of neural activity when participants stay still during scanning. For example, the amplitude of low-frequency fluctuation (ALFF) algorithm of blood-oxygen-level-dependent (BOLD) signal is associated with spontaneous neural activity. The results indicated that ESRD patients exhibited decreased ALFF values in default mode network regions compared with the healthy subjects, suggesting that ESRD patients demonstrated serious spontaneous neural activity abnormalities (Luo et al., 2016). In addition, a recent study demonstrated that hemodialysis patients exhibited decreased regional homogeneity (ReHo) brain regions, notably including bilateral precuneus, posterior cingulate cortex, inferior parietal lobe, right postcentral gyrus, bilateral superior temporal gyri, right supramarginal gyrus, and right angular gyrus. Specific ReHo areas indicated a correlation with cognition impairment, the levels of serum creatinine and urea, and the duration of dialysis (Chen et al., 2015). A total of nine brain regions with ReHo reduction from those mentioned above were defined as seeds for functional connectivity analysis and an increased seed-to-seed functional connectivity was noted [between posterior cingulated cortex (PCC) and precuneus, as well as between PCC and right inferior parietal lobe, PCC and right angular gyrus, and PCC and supramarginal gyrus] in ESRD patients compared with that of the healthy subjects. It is important to note that voxel-based morphometry (VBM) is a method for reflecting the total brain or total intracranial volume (TIV) changes or for detecting the regional alterations of gray matter volume (GMV) based at the voxel level. Widely decreased GMV regions (bilateral occipital lobes, bilateral lingual lobes, bilateral calcarine, precuneus/posterior cingulate cortex/cuneus, bilateral fusiform, right frontal lobe, bilateral superior temporal gyri, bilateral temporal pole, left hippocampus/parahippocampus, left insula, bilateral uncus, right parahippocampus, right amygdala) were found in the ESRD patients compared with the healthy controls as reported by Zhang et al. (2013). Prohovnik et al. (2007) reported that ESRD patients also demonstrated significant brain atrophy, notably in the bilateral caudate nuclei. The morphological brain changes defined as the seed regions may lead to the alterations of functional connectivity in hemodialysis patients. Therefore, the combination of VBM with functional connectivity analysis can investigate the effects of regional GMV changes on whole brain functional integrity and provide simultaneous monitoring of the structural and functional brain alterations, which may be a potential method to explore the neurobiological mechanisms of hemodialysis patients.

Thus, the aims of the present study were three aspects: we sought to detect whether hemodialysis patients had GMV alterations using the VBM method and, if so, we considered these significant structural brain areas as seed regions of interest (ROIs) for cerebral functional connectivity alterations using the functional connectivity analysis; and then we explored the relationship between significantly different regions and clinical variables using the Pearson’s correlation analysis.

## Materials and Methods

### Study Participants

Hemodialysis patients from the Department of Nephrology of Beijing Friendship Hospital and healthy subjects from the local community were enrolled in the present study. A total of 58 hemodialysis patients and 53 control subjects were included. The inclusion criteria for both hemodialysis patients and control subjects were the following: (1) age ≥ 18 years and (2) right-handedness. Hemodialysis patients experienced this condition for at least 6 months. The exclusion criteria were the following: (1) neuropsychiatric history; (2) head trauma history; (3) cerebrovascular disease or other systemic diseases; (4) renal transplantation history; (5) MRI contraindications; and (6) poor imaging quality or head motion. The present study protocol was performed in accordance with the Declaration of Helsinki and was approved by the Medical Research Ethics Committee and Institutional Review Board of the Beijing Friendship Hospital. All participants signed informed consents prior to MRI scanning.

### Clinical Evaluation and Laboratory Examination

Demographic characteristics including age and gender (male and female) of all participants were obtained and the clinical records of the patients, including hemodialysis duration (hemodialysis times) and blood pressure (BP; systolic and diastolic pressure) were reviewed. The brachial artery BP of each patient was measured by a fully automated BP monitor. The average value of three consecutive BP measurements over 10 min was obtained prior to MRI scanning, and the hemodialysis patients were at rest. In addition, routine biochemical tests including hemoglobin (g/l), creatinine (μmol/l), calcium and phosphorus (mmol/l), albumin (g/l), blood urea nitrogen (mmol/l), and serum ferritin (ng/ml) were performed on the inter-dialytic interval day prior to the MRI examination and were analyzed at the clinical laboratory of the Beijing Friendship Hospital. No laboratory examinations were performed in healthy subjects.

### MR Imaging Acquisition

All imaging studies were performed at the Department of Radiology of the Beijing Friendship Hospital. All MRI data were acquired using a 3.0-T magnetic resonance scanner (Discovery MR750, General Electric, Milwaukee, WI, United States) equipped with an eight-channel, phased-array head coil. Prior to scanning, the patients wore earplugs to reduce the scanner noise. During scanning, a tight but comfortable sponge pad was used to fix the subject’s head and minimize motion. All subjects were asked to perform the following actions: stay still, have no thoughts, keep their eyes closed, and stay awake.

The high-resolution three-dimensional brain volume T1-weighted imaging exhibited the following parameters: 196 slices; slice thickness = 1 mm (no gap); repetition (TR)/echo time (TE)/inversion time (TI) = 8.492/3.276/450 ms; flip angle (FA) = 15°; matrix = 256 × 256; field of view (FOV) = 24 × 24 cm^2^. The fMRI data were acquired with the following parameters: 28 slices; 200 time-points; slice thickness = 5 mm (1 mm gap); TR/TE = 2000/35 ms; FA = 90°; matrix = 64 × 64; FOV = 24 × 24 cm^2^.

### Structural Data Preprocessing

Voxel-based morphometry analysis was performed with the standard pipeline of a computational anatomy toolbox (CAT12^1^) implemented in the Statistical Parametric Mapping software (SPM12^2^). The CAT12 toolbox was supplied with different modules and the structural data preprocessing was based on VBM analysis that incorporated the following preprocessing steps (Liu et al., 2019): (1) spatial registration of T1-weighted images to a reference brain template; (2) tissue segmentation into gray matter, white matter, and cerebrospinal fluid; and (3) correction of bias-field inhomogeneity. The segmentations were modulated by scaling with the amount of volume changes due to spatial registration, so that the total amount of gray matter in the modulated image remained the same as it would be in the original image. Finally, the modulated gray matter images were smoothed with a 6 mm full-width at half maximum Gaussion kernel.

### fMRI Data Preprocessing

The functional imaging data preprocessing was carried out by the Data Processing Assistant for Resting-State fMRI (DPARSF^3^) advanced edition software package. (1) For each subject, 200 volumes were acquired in total, and the first 10 volumes were excluded. (2) A total of 190 volumes were corrected for the temporal differences between the slices. (3) Following slice time correction, realignment was performed to correct head motion. Only the subjects with head movement less than 3.0 mm and less than 3.0° rotation in any direction were included. Frame-wise displacement (FD) was calculated and the subjects with a mean FD larger than 0.3 were excluded. The mean FD was further compared between hemodialysis patients and healthy subjects for the volume-to-volume differences in head movement. No significant differences (*P* = 0.8138) in the mean FD between the hemodialysis patients (0.0521 ± 0.0062) and the healthy subjects (0.0504 ± 0.0039) were noted and the mean FD was included as a covariate for the next statistical step. (4) To decrease the non-neural-related signal, the nuisance regressions (motion parameters according to the Friston-24 model, the global signal, the white matter and ventricular signal) were extracted and considered as covariates. Bandpass filtering was subsequently performed on the corrected functional images with a frequency range of 0.01–0.08 Hz. (5) In the subsequently normalization step, individual structural images were co-registered to the mean functional image; the transformed structural images were then segmented and spatially normalized to the Montreal Neurological Institute space using the Diffeomorphic Anatomical Registration Through the Exponentiated Lie (DARTEL) algebra technique (Ashburner, 2007). (6) Spatial smoothing was performed on the normalized images with a 6-mm full-width half maximum isotropic Gaussian kernel.

### Functional Connectivity Analysis

We defined the significantly different GMV areas as seed ROIs for the subsequent functional connectivity analysis. The mean time series for the all seed ROIs were extracted, and their correlations with the time series of the voxels within the total-brain cortices were computed to obtain functional connectivity maps. Additionally, Fisher’s *z* transformation was applied for improving the normality of the data distribution (Liu et al., 2017).

### Statistical Analysis

The two-sample *t-*test was performed to detect the differences in age and TIV between the hemodialysis and the control groups. The Chi-square test was performed to demonstrate differences in gender between the two groups. All statistical analyses were performed using SPSS 17.0 (SPSS, Inc., IBM Company, Chicago, IL, United States). A *P*-value lower than 0.05 (*P* < 0.05) was considered for significant differences.

A two-sample *t-*test was performed to investigate the between-group difference in GMV using SPM12 with TIV, age, and gender as covariates. Multiple comparisons were corrected by a voxel-wise familywise error (FWE) correction method with a corrected threshold of *P* < 0.05. In order to investigate whether significant differences were present with regard to the functional connectivity between the hemodialysis and the control groups, the two-sample *t*-test was performed using SPM12 with age, gender, TIV, and mean FD as covariates. A *P*-value lower than 0.001 (initial height threshold) combined with cluster size larger than 110 voxels was considered as statistically significant [cluster-level false discovery rate (FDR) correction] (Chumbley et al., 2010).

The Pearson’s correlation analysis was performed between the significant brain regions detected from the structural and functional analyses and the identification of specific clinical variables (hemodialysis times, BP, hemoglobin, creatinine, calcium, phosphorus, albumin, blood urea nitrogen, and serum ferritin) that were associated with the clinical outcome of the hemodialysis group. The analysis was conducted using the SPSS software and a statistical significance level was set at *P* < 0.05.

## Results

In total, 58 hemodialysis patients and 53 healthy subjects were enrolled in the present study. A total of 18 out of the 111 subjects were excluded due to the following reasons: (1) cerebrovascular disease (one hemodialysis patient with the left middle cerebral artery occlusion; one healthy subject with arteriovenous malformation, one healthy subject with cavernous hemangioma); (2) removal after head motion correction (six hemodialysis patients and one healthy subject with head motion greater than 3.0 mm or 3.0°; three hemodialysis patients and three healthy controls with mean FD higher than 0.3); (3) inadequate brain images (two hemodialysis patients had claustrophobia). Finally, 46 hemodialysis patients and 47 healthy controls were included for analysis.

### Demographic and Clinical Data Results

The characteristic demographic data of the participants are presented in Table 1. No significant differences between the two groups were observed with regard to the parameters age and gender. The results of the hemodialysis time, BP, and blood tests in the hemodialysis patients are also shown in Table 1.

**TABLE 1:** Demographic characteristics of study and control groups.

**Variables**	**HD (*n* = 46)**	**HC (*n* = 47)**	***P*-value**
Age (years)	53.11 ± 1.58	55.57 ± 0.86	0.1263 (*t* = 1.5430)^a^
Gender (male/female)	28/18	22/25	0.1739 (χ ^2^ = 1.8490)^b^
HD duration (HD times)	484.58 ± 186.31	—	
TIV	1488.21 ± 22.92	1508.63 ± 21.42	0.5371 (*t* = 0.6194)^a^
**Blood pressure, mmHg**		—	
Systolic pressure	145.91 ± 20.54	—	
Diastolic pressure	78.39 ± 13.72	—	
**Blood tests^1^**		—	
Hemoglobin (g/l)	114.30 ± 12.15	—	
Creatinine (μmol/l)	935.01 ± 222.41	—	
Calcium (mmol/l)	2.35 ± 0.22	—	
Phosphorus (mmol/l)	1.46 ± 0.55	—	
Albumin (g/l)	43.55 ± 3.06	—	
Blood urea nitrogen (mmol/l)	20.55 ± 4.95	—	
Serum ferritin (ng/ml)	183.41 ± 125.33	—	

*All values were expressed as mean ± standard deviation or number of subjects. ^*a*^Two-sample *t-*test. ^*b*^Chi-square test. ^1^The normal ranges of hemoglobin, creatinine, calcium, phosphorus, albumin, blood urea nitrogen, serum ferritin are 130–175 g/l, 41–111 μmol/l, 2.11–2.52 mmol/l, 0.85–1.51 mmol/l, 40.00–55.00 g/l, 2.60–7.50 mmol/l, 24.00–336.00 ng/ml in Beijing Friendship Hospital, respectively. A statistical significance level was set at *P* < 0.05. Abbreviation: HD, hemodialysis patients; HC, healthy controls; TIV, total intracranial volume.*

### VBM Analysis Results

**FIGURE 1:**
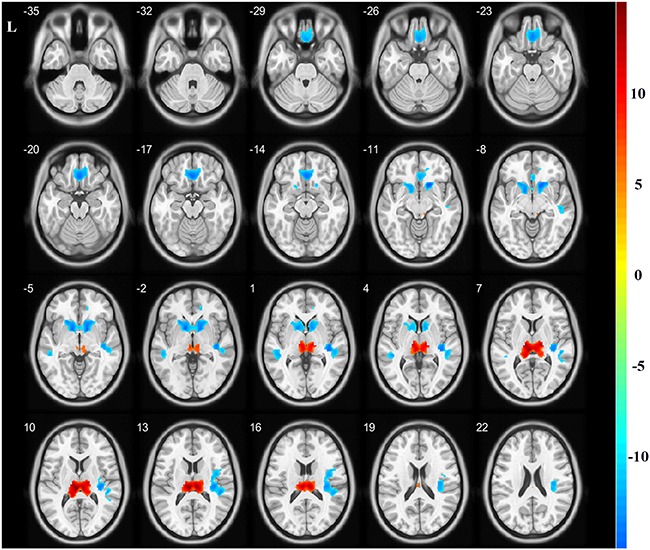
Gray matter volume alterations between the two groups. Compared with the healthy controls, the bilateral thalamus indicated significantly increased volumes in the hemodialysis group compared with those noted in the healthy subjects (hot color). The bilateral rectus, bilateral caudate, and temporal gyrus indicated significantly decreased volumes (cold color). Color bar indicated *t-*value between hemodialysis and healthy control groups. The significance threshold was set at *P* < 0.05 corrected for multiple comparisons with familywise error correction at the voxel level.

A total of five regions indicated significant GMV differences with bilateral location (Figure 1 and Table 2). The bilateral thalamus indicated significantly increased volumes in the hemodialysis patients compared with those noted in the healthy subjects. However, the bilateral rectus, bilateral caudate, and bilateral temporal gyrus demonstrated significantly decreased volumes in the hemodialysis patients (*P* < 0.05, FWE correction).

**TABLE 2:** GMV changes between hemodialysis and healthy control groups.

**Brain regions**	**Peak MNI (mm)**			**Peak *T*-value**	**Number of voxels**
	***x***	***y***	***z***		
Bilateral rectus	–2	32	–16	–7.8179	967
Bilateral caudate	16	18	–10	–8.3287	1152
Bilateral thalamus	–6	–24	12	11.5692	1488
R superior temporal gyrus	40	–26	2	–7.9699	1166
L middle temporal gyrus	–46	–36	4	–6.6231	198

*GMV: gray matter volume; L: left; R: right; MNI: Montreal Neurological Institute; FWE: familywise error. The significance threshold was set at *P* < 0.05 corrected for multiple comparisons with FWE correction at the voxel level.*

### Functional Connectivity Analysis Results

**FIGURE 2:**
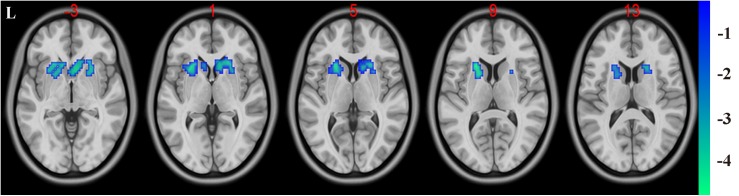
Decreased functional connectivity between the bilateral caudate (defined as seed) and bilateral putamen and caudate. A *P*-value lower than 0.001 (*P* < 0.001) combined with cluster size larger than 110 voxels was considered as statistically significant (cluster-level false discovery rate corrected). Color bar indicated *t*-value between hemodialysis and healthy control groups.

**FIGURE 3:**
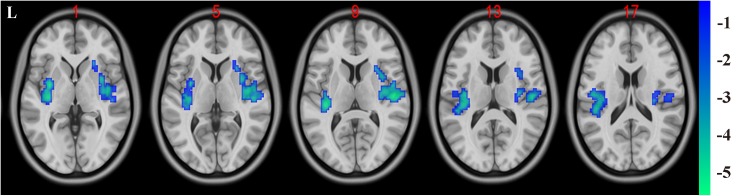
Negative functional connectivity between the bilateral thalamus (defined as seed) and bilateral superior temporal gyrus and insula. A *P*-value lower than 0.001 (*P* < 0.001) combined with cluster size larger than 110 voxels was considered as statistically significant (cluster-level false discovery rate corrected). Color bar indicated *t*-value between hemodialysis and healthy control groups.

In the present study, the GMV alterations of the five significantly different regions were further selected as seed regions for functional connectivity analysis. Therefore, bilateral caudate and thalamus were selected as seed regions and exhibited negative functional connectivity with the bilateral caudate/putamen (see Figure 2 and Table 3) and the bilateral temporal gyrus/insula (see Figure 3 and Table 4), respectively (*P* < 0.05, cluster-level FDR correction). No significant changes in functional connectivity were detected in the other three brain seed regions (bilateral rectus, right and left temporal gyrus).

**TABLE 3:** Significant difference between hemodialysis patients and healthy controls in functional connectivity between the bilateral caudate (used as seed area) and the rest of the whole brain.

**Brain regions**	**Peak MNI (mm)**			**Peak *T*-value**	**Number of voxels**
	***x***	***y***	***z***		
L putamen and caudate	−21	18	9	−5.0059	173
R putamen and caudate	6	15	−3	−4.5817	119

*L: left; R: right; MNI: Montreal Neurological Institute; FDR: false discovery rate. The significance threshold was set at *P* < 0.001 (initial height threshold) combined with cluster size > 110 voxels (cluster-level FDR correction).*

**TABLE 4:** Significant difference between hemodialysis patients and healthy controls in functional connectivity between the bilateral thalamus (used as seed area) and the rest of the whole brain.

**Brain regions**	**Peak MNI (mm)**			**Peak *T*-value**	**Number of voxels**
	***x***	***y***	***z***		
L superior temporal gyrus and insula	−33	−3	0	−5.4265	359
R superior temporal gyrus and insula	51	−18	9	−5.3033	275

*L: left; R: right; MNI: Montreal Neurological Institute; FDR: false discovery rate. The significance threshold was set at *P* < 0.001 (initial height threshold) combined with cluster size > 110 voxels (cluster-level FDR correction).*

### Correlation Between Significant Brain Regions and Blood Test Variables

At the structural level, no significant negative or positive association was noted between the five abnormal GMV regions and the hemodialysis times, the BP, and the levels of the blood test variables examined in the hemodialysis patients. No significant differences were noted between the two groups with respect to the brain functional connectivity, hemodialysis times, BP, and blood data.

## Discussion

The present study reported three main significant findings: (1) VBM analysis demonstrated increased volume in the bilateral thalamus and decreased volume in the bilateral rectus, bilateral caudate, and bilateral temporal gyrus in hemodialysis patients compared with the corresponding parameters noted in healthy subjects. (2) Decreased functional connectivity between bilateral caudate and thalamus as seed areas with the bilateral caudate/putamen and bilateral temporal gyrus/insula, respectively. (3) All significant brain regions were in the bilateral cerebral hemispheres using both VBM analysis and functional connectivity analysis. These findings indicated that GMV and functional connectivity alterations were prevalent in hemodialysis patients, whereas the bilateral distribution of the regions in cerebral hemispheres may be one of the traits noted in hemodialysis patients.

In the current study, the reduction of GMV was presented in the bilateral rectus, the bilateral temporal gyrus, and the bilateral caudate. However, increased GMV in the bilateral thalamus was found in hemodialysis patients compared with the healthy subjects. Rectal gyrus is the one part of the prefrontal cortex (PFC) and the reduction of GMV in the PFC of hemodialysis patients detected in our study was consistent with the findings reported in previous studies. The hemodialysis patients exhibited GMV reduction in several regions including the bilateral medial orbitofrontal cortex, the bilateral dorsolateral PFC, and the left middle temporal gyrus, as reported by Qiu et al. (2014). Ma et al. (2014) further showed that the GMV of the bilateral PFC, temporal gyrus, anterior cingulate gyrus, and other widespread regions were decreased in hemodialysis patients. In addition to GMV reductions in PFC, metabolism abnormalities were also observed using the positron emission tomography method (Luo et al., 2016). Significantly decreased cerebral glucose metabolism has been noted in several regions including the bilateral PFC, the left anterior cingulate gyrus, the left temporal gyrus, and other crucial brain regions. Moreover, a negative correlation was reported between the cerebral glucose metabolism of the right PFC and the Hamilton Depression Rating Scale in chronic kidney disease patients. To the best of our knowledge, PFC is involved in executive functions of higher-level cognitive processing, as well as in language, emotional processing, and social skills. Therefore, it serves as the most critical region in the cortical area required for executive function, such as the processing of diverse sensory modalities, attention, social judgment, working memory, abstract thinking, and intentionality (Teffer and Semendeferi, 2012). The GMV reduction in PFC detected in the present study was in accordance with that noted in previous studies and may be associated with the executive function deficits. This condition may be the prominent feature noted in hemodialysis patients.

A previous study suggested that hemodialysis patients displayed decreased *N*-acetylaspartate and creatine ratio (NAA/Cr) in the thalamus compared with that of control subjects, which played an important role in neuropathological mechanisms and revealed brain functional impairment in hemodialysis patients (Ma et al., 2018). Furthermore, Chai et al. (2015) demonstrated a strong positive correlation between the susceptibility of the thalamus and the hemodialysis duration, using susceptibility weighted imaging analysis. This study suggested that the longer dialysis duration could lead to increased iron deposition in the brain of hemodialysis patients. In the present study, it was shown that bilateral thalamus exhibited significantly increased volumes in the hemodialysis patients compared with those of the healthy subjects, whereas no significant correlation was noted between the clinical features (hemodialysis times, BP, and blood examination variables) and the volume alterations of the thalamus. The aforementioned neuroimaging studies investigated the thalamic alterations in hemodialysis patients by different imaging methods. Following investigation of the regional alterations in the GMV, significant reductions were noted in the bilateral caudate and other brain regions of the hemodialysis patients in the present study. However, in a previous study by Prohovnik et al. (2007) decreased GMV was only present in the bilateral caudate and not in other areas. The most likely reason for the difference noted in our study and that of Prohovnik et al. was that the latter included only 10 hemodialysis patients and six healthy subjects. This demonstrated that bilateral caudate atrophy in these patients could be one of the fundamental anatomical mechanisms associated with this hemodialysis.

Despite the aforementioned studies focused on GMV of the PFC, the temporal gyrus, bilateral thalamus, and caudate, no brain area functions as a separated or isolated entity. The discussion of the functional connectivity was based on the regions as seeds detected from the VBM analysis indicating the extensive interconnections with other brain regions. In the present study, it was shown that when the seed was located in the bilateral caudate, the hemodialysis patients indicated reduced functional connectivity in the bilateral putamen and caudate, suggesting impairments of basal ganglia (BG) circuitry in ESRD patients with hemodialysis. The BG system comprises the BG and the connections between BG and the cortical areas (Schmitt et al., 2016). The BG nuclei play a key function in several neuronal pathways, including the emotional, motivational, and cognitive functions. The cortico-striatal connectivity has been generally considered a rich set of pathways between the putamen and the caudate with frontal and parietal regions. Previous findings have shown that caudate is principally connected with the frontal cortex, such as the ventral PFC, superior frontal gyrus, rostral middle frontal gyrus, and orbitofrontal gyrus (Cacciola et al., 2017). In contrast to the latter study, the data presented in the current report revealed decreased intra-basal-ganglia-connection and absence of suprathreshold connections in the frontal or parietal area with caudate. The most likely reason for the inconsistent results noted in the present study may be the small sample size of the cohort. Furthermore, the present study exhibited heterogeneous clinical features, such as the hemodialysis duration and modalities, which may have affected the results. Although the role of decreased intra-basal-ganglia-connection in hemodialysis patients has not been fully explored, the results of the present study provided novel insight into the underlying mechanism of the hemodialysis patients. In addition, when the seed was located in the bilateral thalamus, the hemodialysis patients indicated reduced functional connectivity in the bilateral superior temporal gyrus and insula, suggesting thalamo-cortical network hypo-connectivity. To our knowledge, bilateral thalamus that is defined as seed regions for functional connectivity analysis in hemodialysis patients has not yet been thoroughly investigated although it is meaningful for understanding the neural mechanisms in hemodialysis patients. The thalamus is a major sensory conduction relay station in the brain that plays a filtering role as in the thalamo-cortical network for cortical sensory input regulation and excitability control (Borsook, 2012). Therefore, the present study demonstrated that the increased GMV in the bilateral thalamus may reflect central sensitization due to the increased number of neurons, suggesting additional sensory signal input. The thalamus regulates the processing of motivation, emotion, planning, and expression of goal-directed behaviors. Furthermore, previous studies have demonstrated the key role of the insular in pain-associated diseases, such as headache (Zheng et al., 2016), suggesting that it is one part of the pain sensory system responsible for receiving direct projections from the thalamus. The insular mainly accepts pain perception and it can relieve pain (Brooks and Tracey, 2007). In addition, more than 50% of hemodialysis patients experience pain, approximately 69% of hemodialysis patients present with joint pain, and almost all of the patients with hemodialysis therapy exhibit non-specific symptoms, such as cramps or headaches (Davison, 2003), resulting from alterations in the concentration of metabolic substances, such as dopamine receptors in the insular (Ferrier et al., 2015). Therefore, decreased thalamo-insular connection may be an important mechanism of pain in hemodialysis patients, suggesting that insular is a key brain region in pain perception. Furthermore, the present study revealed the presence of thalamo-temporal hypo-connectivity in hemodialysis patients, which may be due to impaired thalamo-cortical modulation underlying emotional disturbances. This is in line with previous neuroimaging studies in major depressive disorders (Milak et al., 2005; Yamamura et al., 2016). Nevertheless, thalamo-cortical networks may be a potential candidate marker for psychopathology in hemodialysis patients. Finally, the present study highlighted that structural and functional brain alterations were located in bilateral cerebral hemispheres. This finding may be attributed to the following reasons: (1) ESRD is a systematic and metabolic disease involving brain structural and functional changes. (2) The cerebrum and glomerulus are closely connected with each other through the “kidney–brain axis,” which is responsible for anatomical (Bugnicourt et al., 2013) and pathological changes (Masaki and Masatsugu, 2010).

In addition, the current data failed to report on significant negative or positive associations between brain regions, detected from structural and functional analyses, and clinical data of hemodialysis times, BP, and blood examinations performed in hemodialysis patients. However, only hemoglobin levels were slightly below the normal range and creatinine and blood urea nitrogen concentration levels were substantially higher than the normal range due to the final stage of chronic kidney disease. Hemodialysis patients always suffered from anemia, malnutrition, and protein-energy wasting conditions that resulted from different pathogenic causes. Previous studies have shown that hemodialysis patients are usually vulnerable to under-nutrition problems caused by insufficient nutrient supply to the brain, resulting in brain abnormalities, such as cerebral hypoxia, decreased blood viscosity, brain hypo-perfusion, and/or hypo-metabolism (Yasuo et al., 2002; Christina et al., 2012). Therefore, long-term hemodialysis can significantly affect the cerebral circulation, function, and metabolism, leading to poor mental health of hemodialysis patients due to low hemoglobin levels (Christina et al., 2012). Other laboratory biochemical indicators exhibited a relatively normal range and it was speculated that hemodialysis patients on regular use of vitamin D, calcitriol, and/or phosphorus-chelating agents might have contributed to the steady state noted in the present study.

The present study contains several limitations. Initially, the effects of different dialysis modalities were not assessed regarding structural and functional brain alterations, since the exact way and the time point by which hemodialysis modalities affected the brain was unknown. Nevertheless, brain changes in ESRD patients on hemodialysis may be associated with cognitive deficits. In the current study, the abnormal GMV/functional connectivity did not exhibit a significant correlation with clinical variables (hemodialysis times and BP) and blood examination parameters and therefore a more detailed investigation is required in future studies. Moreover, Pearson correlation analysis was not performed between neuropsychology tests and GMV alterations/functional connectivity, which could potentially improve our understanding of the neuropsychological mechanisms of the ESRD patients on hemodialysis. In addition, it was shown that when the seed was in the bilateral caudate, the hemodialysis patients indicated reduced functional connectivity in the bilateral putamen and caudate, similar to the impairments of intra-network connection in BG circuity. The result provided at least imaging evidence of brain changes in the hemodialysis patients. However, we cannot rule out that signals obtained from caudate comprise fewer functional voxels and thus are influenced by noise. Future studies with other reproducibility validations are required for identifying the measurement artifact. Lastly, the present study exhibited a small sample size and future studies with larger sample sizes are required.

## Conclusion

In conclusion, the present study performed VBM analysis and functional connectivity to monitor the structural and functional alterations in hemodialysis patients. The findings revealed that hemodialysis patients demonstrated GMV alterations in several regions and that these regions, which were defined as seeds, exhibited significant hypo-connectivity in the thalamo-cortical network and within the basal-ganglia connection. The present study revealed different types of structural and functional brain impairment in hemodialysis patients.

## Data Availability Statement

All datasets generated for this study are included in the article/supplementary material.

## Ethics Statement

The studies involving human participants were reviewed and approved by the Medical Research Ethics Committees and Institutional Review Board of Beijing Friendship Hospital. The patients/participants provided their written informed consent to participate in this study.

## Author Contributions

MJ, HW, LW, XH, ZW, PZ, WG, ZD performed the experiment and collected, analyzed, or interpreted the data involved in the study. MJ preprocessed image data, performed the statistical results, and drafted the manuscript. WL, ZCW, PFZ, HL, ZY, and HD designed the study and ensured the questions related to all aspects of the work. ZCW, WL, MJ, and HW gave critical comments on the manuscript.

## Conflict of Interest

The authors declare that the research was conducted in the absence of any commercial or financial relationships that could be construed as a potential conflict of interest.

## Footnotes

1 http://www.neuro.uni-jena.de/cat/

2 https://www.fil.ion.ucl.ac.uk/spm/

3 http://rfmri.org/dpabi
